# Performance of ICU ventilators during noninvasive ventilation with large leaks in a total face mask: a bench study* **

**DOI:** 10.1590/S1806-37132014000300013

**Published:** 2014

**Authors:** Maria Aparecida Miyuki Nakamura, Eduardo Leite Vieira Costa, Carlos Roberto Ribeiro Carvalho, Mauro Roberto Tucci

**Affiliations:** Laboratório de Investigação Médica 09 (LIM 09, Laboratory for Medical Research 09), Department of Pulmonology, Heart Institute, University of São Paulo School of Medicine Hospital das Clínicas, São Paulo, Brazil; Respiratory ICU, Department of Pulmonology, Heart Institute, University of São Paulo School of Medicine Hospital das Clínicas; and Researcher, Instituto de Ensino e Pesquisa, Hospital Sírio-Libanês, São Paulo, Brazil; Department of Cardiorespiratory Diseases, University of São Paulo School of Medicine, São Paulo, Brazil; Laboratório de Investigação Médica 09 (LIM 09, Laboratory for Medical Research 09), Department of Pulmonology, Heart Institute, University of São Paulo School of Medicine Hospital das Clínicas, São Paulo, Brazil

**Keywords:** Ventilators, mechanical, Positive-pressure Respiration, Noninvasive ventilation, Equipment safety, Equipment failure, Masks

## Abstract

**Objective:**

Discomfort and noncompliance with noninvasive ventilation (NIV) interfaces are obstacles to NIV success. Total face masks (TFMs) are considered to be a very comfortable NIV interface. However, due to their large internal volume and consequent increased CO_2_ rebreathing, their orifices allow proximal leaks to enhance CO_2_ elimination. The ventilators used in the ICU might not adequately compensate for such leakage. In this study, we attempted to determine whether ICU ventilators in NIV mode are suitable for use with a leaky TFM.

**Methods:**

This was a bench study carried out in a university research laboratory. Eight ICU ventilators equipped with NIV mode and one NIV ventilator were connected to a TFM with major leaks. All were tested at two positive end-expiratory pressure (PEEP) levels and three pressure support levels. The variables analyzed were ventilation trigger, cycling off, total leak, and pressurization.

**Results:**

Of the eight ICU ventilators tested, four did not work (autotriggering or inappropriate turning off due to misdetection of disconnection); three worked with some problems (low PEEP or high cycling delay); and one worked properly.

**Conclusions:**

The majority of the ICU ventilators tested were not suitable for NIV with a leaky TFM.

## Introduction

Noninvasive ventilation (NIV) has been used successfully in order to manage respiratory failure of different etiologies.^(^ ^1^ ^)^ Adherence to treatment is a major concern and has a profound impact on NIV success. In acute respiratory failure, for example, 40-60% of NIV trials fail due to mask discomfort and patient noncompliance.^(^ ^2^ ^-^ ^4^ ^)^

The total face mask (TFM) is an alternative interface designed to increase patient tolerance. It covers the entire face, delivering effective ventilation via nasal and oral routes. By means of its increased contact surface with the skin, it also minimizes gas leakage whilst avoiding pressure sores to the face.^(^ ^5^ ^-^ ^7^ ^)^ However, TFM has the disadvantage of having a large internal volume (875 mL).^(^ ^8^ ^)^ Therefore, in order to minimize CO_2_ rebreathing, the mask has two built-in exhalation ports that allow air leakage.^(^ ^5^ ^,^ ^9^ ^,^ ^10^ ^)^ These intentional air leaks are often adequately compensated by NIV ventilators but might not be handled as well by ICU ventilators.^(^ ^11^ ^)^

The main hypothesis of our study was that ICU ventilators do not perform adequately with TFM with large air leaks. We evaluated the performance of ICU ventilators in delivering NIV via TFM with large air leaks on a bench model and compared the results obtained with a mechanical ventilator dedicated for NIV.

## Methods

This was an experimental study conducted in 2008 in the Laboratory for Medical Research 09, specializing in Pulmonology, at the University of São Paulo School of Medicine, in the city of São Paulo, Brazil.

The NIV model (Figure E1, available online at http://www.jornaldepneumologia.com.br/imagebank/images/jbp_v40n3_suplemment.pdf) was adapted from previously described models^(^ ^12^ ^,^ ^13^ ^)^ and consisted of a two-chamber test lung (TTL 2600; Michigan Instruments, Grand Rapids, MI, USA) partially connected by a lift bar. The first chamber (drive chamber) was connected to the drive ventilator and, during the inspiratory phase, was insufflated, moving the second chamber (chest chamber) together and producing a negative pressure in its interior, which was transmitted to a second one-chamber test lung (Takaoka, São Paulo, Brazil). This simulator consists of a bellows device in a rigid box, the bellows representing the "lung", and the space between the bellows and the box representing the pleural space with direct communication with the chest chamber. The "lung" was connected to a mannequin head made of PVC. A TFM (Philips Respironics, Murryville, PA, USA) was attached to the mannequin head and connected to the test ventilator. The mechanical lung model compliance was 50 mL/cmH_2_O at an inspiratory volume of 500 mL.

Two pressure transducers (Valydine, Northridge, CA, USA) were connected to the model (Figure E1): one for proximal pressure (between the mask and the proximal pneumotachograph) and one for pleural pressure (between the two-chamber test lung and the one-chamber test lung). The flow was measured with two pneumotachographs (Hans-Rudolph, Kansas City, MO, USA): one for proximal flow (between the proximal pressure transducer and the Y connector from the ventilator circuit) and one for distal flow (between the upper airway of the mannequin and the one-chamber test lung). The resistance of the proximal pneumotachograph varied with the flow values. For flows of 0.5 L/s, 1.0 L/s, 2.0 L/s, and 3.0 L/s, resistance was 1.29 cmH_2_O . L^−1^ . s^−1^, 1.44 cmH_2_O . L^−1^ . s^−1^, 1.91 cmH_2_O . L^−1^ . s^−1^, and 2.40 cmH_2_O . L^−1^ . s^−1^, respectively. The analogical signals from the transducers were recorded at 200 Hz and analyzed off-line with a customized Labview software program (National Instruments, Austin, TX, USA).

A Newport e500 ventilator (Newport Medical Instruments, Costa Mesa, CA, USA) was used in order to provide the inspiratory effort for the NIV model. Respiratory rate was 12 breaths/min in pressure control mode. Inspiratory time was 1.0 s, driving pressure was 17 cmH_2_O, positive end-expiratory pressure (PEEP) was 0 cmH_2_O, and inspiratory slope was +2, developing a tidal volume in the mechanical lung model of 300 mL and an airway occlusion pressure after 0.1 s (P0.1) of 3.4 cmH_2_O.

### Ventilators tested

One NIV ventilator (BiPAP Vision; Philips Respironics) and eight ICU ventilators, all equipped with noninvasive mode, were tested (Table E1, available online at http://www.jornaldepneumologia.com.br/imagebank/images/jbp_v40n3_suplemment.pdf): Puritan Bennett 840 (Covidien, Boulder, CO, USA); Servo-i (Maquet, Solna, Sweden); Vela (Viasys Healthcare, Palm Springs, CA, USA); Savina (Drägerwerk AG & Co., Lübeck, Germany); Esprit (Philips Respironics); GALILEO Gold (Hamilton Medical, Rhäzuns, Switzerland); Horus (Taëma, Anthony, France); and e500 (Newport Medical Instruments).

Whenever available, the pressure trigger was used and set to the most sensitive level that did not result in autotriggering. When the pressure trigger was unavailable or autotriggering was unavoidable, we used the flow trigger. When adjustable, inspiratory rise time was set, initially, at 50% of the maximum value, expiratory threshold was set at 25% of peak inspiratory flow (PIF), and maximum inspiratory time was set at 1.5 s. The evaluations were performed using pressure support model, with PEEPs of 5 cmH_2_O (PEEP5) and 10 cmH_2_O (PEEP10), each with three different pressure support levels: 5 cmH_2_O (PS5); 10 cmH_2_O (PS10); and 15 cmH_2_O (PS15). Initially, each ventilator was tested with a sealed facial mask in order to verify the NIV functionality of the ventilator. The TFM was subsequently tested with the two exhalation ports open to allow air to escape, as recommended by the manufacturer.^(^ ^5^ ^)^

### Measured variables

First, we evaluated whether each ventilator worked properly with the TFM. The "no operation" of the ventilator was defined as the presence of constant autotriggering or of the inspiratory flow turning off (misinterpretation of disconnection due to massive leakage) even after trying different settings of triggering and inspiratory rise time. If the ventilator worked, we recorded the additional adjustments necessary to make it work properly. 

**Figure 1:**
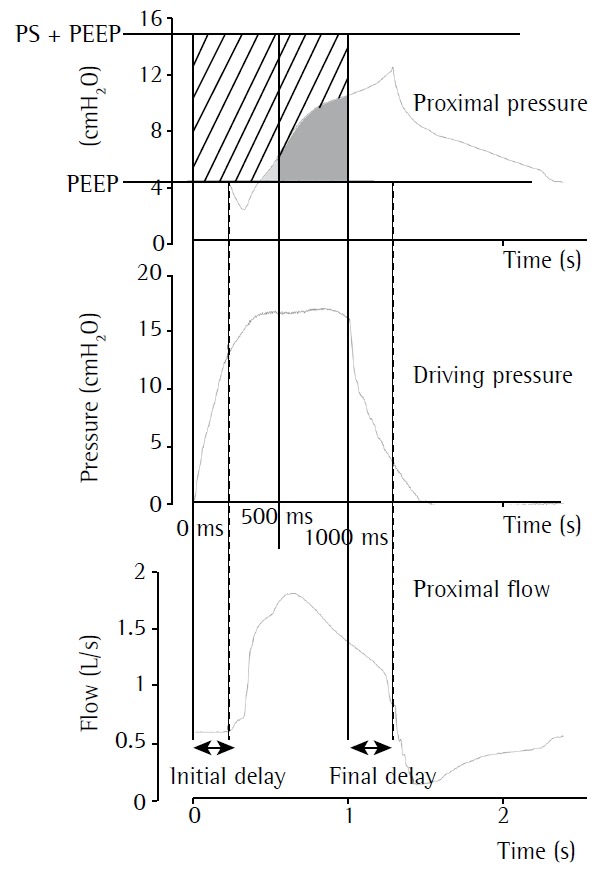
Representation of the variables measured. Pressure (upper) and flow (lower) tracings of a hypothetical measurement with positive end-expiratory pressure (PEEP) of 5 cmH_2_O and pressure support (PS) of 10cmH_2_O. In the middle, tracing of the pressure for the drive ventilator. The upper tracing shows the inspiratory pressure-time product at 500 ms (PTP500; light gray area) and at 1 s (PTPt; dark gray area plus light gray area), both expressed in percentage of ideal area (line-shaded areas)

We measured the following variables (Figure 1): proximal inspiratory pressure (PIP) at the end of the inspiratory phase, in cmH_2_O, measured at the proximal sensor; PEEP, in cmH_2_O; inspiratory leakage, in L/s, determined by the difference between proximal flow and distal flow at PIF; expiratory leakage, in L/s, by measuring proximal flow at the end of the expiratory phase; PIF, in L/s; tidal volume, in L, calculated by the integration of the flow signal from the distal flow transducer; trigger delay, in ms, determined by the time elapsed between the onset of inspiratory effort (in pleural pressure) and the onset of inspiratory flow; cycling-off delay, in ms, measured by the time from the end of the driving inspiratory effort to the end of the ventilator inspiratory flow; and inspiratory pressure-time product at 500 ms and at 1 s (PTP500 and PTPt, respectively), determined by computing the area under the pressure-time curve between the onset of inspiratory effort and these two times.

### Statistical analysis

For each experimental condition, the software calculated a representative mean cycle through a point-by-point averaging of five cycles. Data for each variable are shown as one value (from the mean cycle) for each condition. Data for the various conditions are shown as mean ± SD.

The cycles were very stable, and the variances for each variable were negligible; therefore, in our analysis, we did not use formal statistical hypothesis tests but a nominal comparison of the values of each mean cycle, such as in other similar studies.^(^ ^13^ ^,^ ^14^ ^) ^Because of the low variability of the NIV mechanical model, any clinically relevant difference in the measurements would certainly result in a difference of more than 2.8 times the measurement error (standard deviation of various cycles of one condition).^(^ ^15^ ^)^ The calculated values (2.8 times the measurement error) were as follows: tidal volume, 0.020 L; PIP, 0.17 cmH_2_O; PEEP, 0.15 cmH_2_O; PTP500, 4.3%; PTPt, 2.5%; PIF, 0.05 L/s; expiratory leak, 0.03 L/s; trigger delay, 9.7 ms; and cycling-off delay, 7.5 ms.

## Results

The NIV ventilator and only four of the ICU ventilators equipped with NIV mode worked with the TFM: Horus, Vela, e500, and Servo-i (Table 1). Of these, all but the Servo-i had problems to trigger or to cycle off. The main problems in the other ventilators, which were considered to be non-operational, were the misinterpretation of disconnection and autotriggering. With the sealed oronasal mask (i.e., in the absence of significant system leakage), all ventilators worked well.

**Table 1:** - Performance of the ventilators testeda with the total face mask and the major problems observed.

Ventilator	Proper operation	Cause of no operation	Problems during operation
BiPAP Vision	Yes		
Puritan Bennett 840^b,c^	No	AT	
Savina^b,d^	No	AT	
GALILEO Gold^b,e^	No	FTO	
Servo-i^b,f^	Yes		Premature cycling at PEEP5 and PS5 and at PEEP10 and PS5
e500^b,e^	No	FTO (nonoperational at PEEP10)	LCF at PEEP5 and the 3 PS settings
Esprit^b,g^	No	AT, FTO	
Horus (NIV deactivated)	No	FTO (nonoperational at PEEP5)	CIT and LCF at PEEP10 and the 3 PS settings
Horus^b,e^	No	AT	
Vela^b,h^	No		CIT

NIV : noninvasive ventilation

AT: autotriggering (the ventilator tested maintained a respiratory rate larger than that of the drive ventilator (12 breaths/min) and the inspiratory time was variable)

FTO : inspiratory flow turning off (inappropriate turning-off after some cycles due to misinterpretation of disconnection)

PS5 : pressure support of 5 cmH_2_O

**PEEP5:** : positive end-expiratory pressure of 5 cmH_2_O

**PEEP10:** : positive end-expiratory pressure of 10 cmH_2_O

LCF : leakage compensation failure (ventilator cannot maintain PEEP level)

CIT : cycling by maximum inspiratory time adjusted in 1.5 s

a BiPAP Vision (Philips Respironics, Murryville, PA, USA); Puritan Bennett 840 (Covidien, Boulder, CO, USA); Savina (Drägerwerk AG & Co., Lübeck, Germany); Galileo Gold (Hamilton Medical, Rhäzuns, Switzerland); Servo-i (Maquet, Solna, Sweden); e500 (Newport Medical Instruments, Costa Mesa, CA, USA); Esprit (Philips Respironics); Horus (Taëma, Anthony, France); and Vela (Viasys Healthcare, Palm Springs, CA, USA)

b Noninvasive ventilation activated. Updates on ventilator capabilities between 2008 and 2013

c Puritan Bennett(tm) has a new optional software with leak compensation up to 65 L/min for adults

d No change in leak compensation. The new model (Savina 300) has improved leak compensation

e No updates for leak compensation

f The new version of the NIV mode compensates for leaks up to 65 L/min for adults

g The new software version and new model (V200) have leak compensation up to 60 L/min and autoadaptative triggering and cycling-off (autotracking)

h Leak compensation up to 40 L/min in Vela Plus and Vela Comprehensive models

In all ICU ventilators, except for the Horus, we needed to turn the NIV mode on. For the Horus, e500, and Vela to work in some settings, we needed to adjust the expiratory trigger sensitivity, the inspiratory pressure slope, or the inspiratory sensitivity.

The Horus ventilator did not work in NIV mode. Using the default "invasive mode", we had to set it to pressure support mode, with its expiratory cycling threshold and inspiratory pressure-slope adjusted to maximum (30 L/min and 150 cmH_2_O/s, respectively). Inspiratory trigger sensitivity could not be properly adjusted with the pressure-trigger option and worked only within a narrow range with the flow-trigger option (approximately 1.7 L/min).

The e500 inspiratory pressure slope was set to automatic mode, as was the expiratory cycling threshold at 50% of peak flow. To avoid autotriggering, it was necessary to set the pressure trigger at values close to −3.2 cmH_2_O.

The Vela NIV mode has an automatic selection of inspiratory triggering sensitivity. Expiratory cycling threshold had to be set at maximum (30% of peak flow). However, when PEEP was set at 10 cmH_2_O and pressure support was set at 15 cmH_2_O, there was a high inspiratory cycling-off delay, creating large tidal volumes that exceeded the lung model capacity.

The Servo-i worked properly. Its NIV mode has automatic inspiratory triggering sensitivity. The inspiratory pressure slope was adjusted to 50% of maximum (0.2 s), and the expiratory cycling threshold was set at 25% of peak flow.

The inspiratory flow slope of the NIV ventilator (BiPAP Vision) was adjusted to 50% of maximum (0.2 s). The inspiratory-triggering and expiratory-triggering sensitivity were automatic.

**Figure 2:**
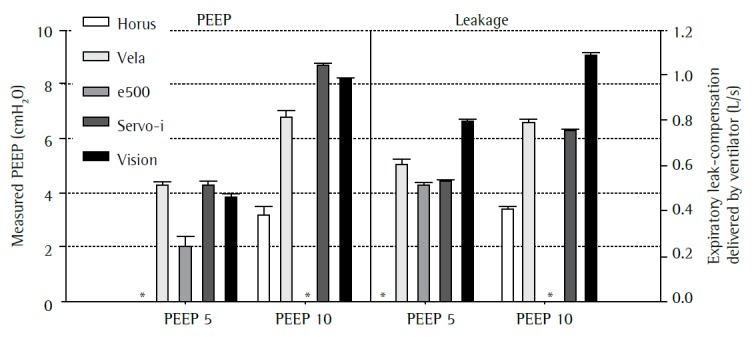
Measured positive end-expiratory pressure (PEEP; left panel) and expiratory flow delivered by the ventilator (right panel) to compensate for air leakage at PEEP of 5 cmH_2_O (PEEP5) and 10 cmH_2_O (PEEP10), expressed as mean ± SD. As shown, the Horus and the e500 ventilators did not compensate adequately for leaks, delivering less than 0.6 L/s of compensatory flow, and were not capable of keeping the set PEEP level. *Not measured due to autotriggering.

Regarding PEEP and expiratory leakage, the Servo-i, Vela, and BiPAP Vision compensated for the TFM leaks during exhalation, maintaining PEEP values close to the set values (Figure 2). The mean flows delivered by these ventilators in order to compensate for leaks during exhalation were 0.65 ± 0.12 L/s and 0.89 ± 0.16 L/s for PEEP5 and PEEP10, respectively. The e500 and Horus compensated for the leakage poorly and therefore were not able to maintain the target PEEP (Figure 2). The maximum leakage-compensation flows for the e500 and Horus were, respectively, 0.52 ± 0.01 L/s (PEEP5 with leakage compensation option switched on) and 0.41 ± 0.01 L/s (PEEP10).

**Figure 3:**
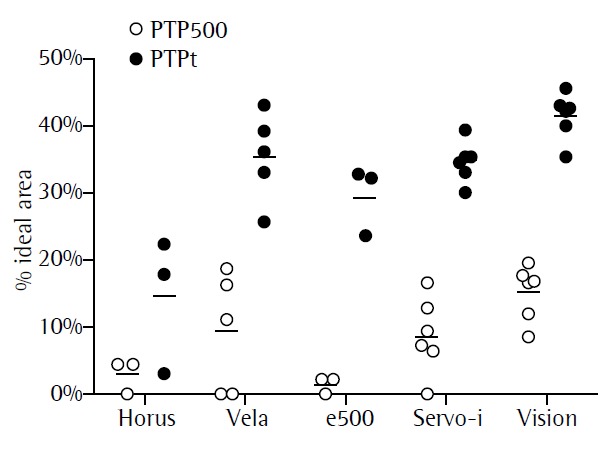
Pressurization characteristics of five of the ventilators tested, demonstrated by inspiratory pressure-time product at 500 ms (PTP500; open circles) and at 1 s (PTPt; filled circles), expressed in percentage of ideal area. The horizontal dashes indicate the means of all measures (positive end-expiratory pressures of 5 and 10 cmH_2_O vs. pressure support of 5, 10 and 15 cmH_2_O) available for the ventilators.

The Horus ventilator had the lowest PIP at the mask (Figure E2, available online at http://www.jornaldepneumologia.com.br/imagebank/images/jbp_v40n3_suplemment.pdf). All of the other ventilators reached similar PIPs. The BiPAP Vision presented with the highest PTP500 and PTPt values (Figure 3). The e500 and Horus had the lowest values for PTP500.

**Figure 4:**
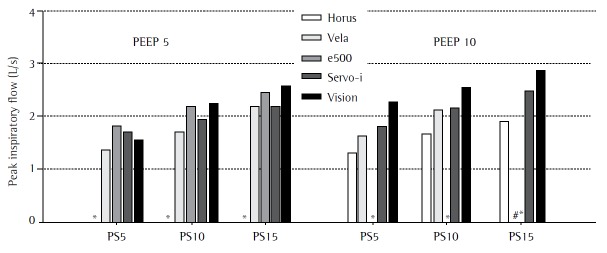
Peak inspiratory flow of five of the ventilators tested. Measurements with positive end-expiratory pressure of 5 cmH_2_O (PEEP5) and 10 cmH_2_O (PEEP10) are on the left and right sides, respectively. The measurements were taken at pressure support levels of 5, 10, and 15 cmH_2_O (PS5, PS10, and PS15, respectively). *The Horus ventilator at PEEP5 and the e500 ventilator at PEEP10 were not measured due to autotriggering. #Because of the limitation of the lung model, the Vela ventilator was not tested for PEEP10 with PS15.

The PIF values increased in parallel with increases in inspiratory pressures (Figure 4), and the volume of leaks increased in parallel with increases in PEEP and pressure support. The mean values of PIF for all ventilators were 1.69 ± 0.31 L/s, 2.07 ± 0.26 L/s, and 2.36 ± 0.31 L/s, respectively, at PS5, PS10, and PS15. The highest PIFs were reached by the BiPAP Vision and e500 (2.39 ± 0.32 L/s and 2.14 ± 0.32 L/s, respectively). The Servo-i and the Vela ventilators had intermediate values (2.00 ± 0.32 L/s and 1.82 ± 0.33 L/s, respectively), and the smallest PIFs were attained by the Horus (1.60 ± 0.29 L/s).

Regarding tidal volumes, they became higher with the increase in pressure support levels (Figure E3, available online at http://www.jornaldepneumologia.com.br/imagebank/images/jbp_v40n3_suplemment.pdf). The mean tidal volumes for all ventilators using PEEP5 at PS5, PS10, and PS15 were 472 ± 25 mL, 609 ± 32 mL, and 726 ± 124 mL, respectively, whereas the mean tidal volumes for the ventilators using PEEP10 at the same three pressure support modes were 489 ± 25 mL, 641 ± 28 mL, and 768 ± 90 mL, respectively. 

The mechanical lung model (without the test ventilators) had an intrinsic baseline delay of 30.5 ms and 24.9 ms, respectively, for trigger delay and cycling-off delay. Only the e500 and Horus presented with trigger delay values higher than 100 ms (276 ± 105 ms and 152 ± 36 ms, respectively). The Servo-i, Vela, and BiPAP Vision, respectively, had trigger delay values of 50 ± 12 ms, 53 ± 6 ms, and 69 ± 27 ms (Figure E4, available online at http://www.jornaldepneumologia.com.br/imagebank/images/jbp_v40n3_suplemment.pdf). Neither PEEP nor pressure support altered the triggering delay. Although the Servo-i ventilator had the lowest cycling-off delay (8.5 ± 67 ms), it presented with premature cycling in some settings (−94 ms for PEEP5 at PS5, as well as −43 ms for PEEP10 at PS5). The BiPAP Vision ventilator had a mean cycling-off delay of 136 ± 92 ms. The Horus and Vela had mean values of 273 ± 231 ms and 228 ± 214 ms, respectively. In both, cycling sometimes occurred by reaching the adjusted maximum inspiratory time of 1.5 s. The e500 ventilator showed the highest cycling-off delay (590 ± 622 ms; Figure E5, available online at http://www.jornaldepneumologia.com.br/imagebank/images/jbp_v40n3_suplemment.pdf).

## Discussion

The most important finding in the present study was that only one of the tested ICU ventilators was suitable for NIV using a TFM. Of the eight ICU ventilators, four were considered totally non-operational due to inappropriate turning-off (misinterpretation of disconnection) or autotriggering, whereas three of the remaining four had problems to compensate for the large leaks through the exhalation ports, resulting in inability to keep PEEP and inspiratory pressure, delayed inspiratory triggering, or delayed inspiration-to-expiration cycling. Only the Servo-i and the control NIV ventilator (BiPAP Vision) worked properly under all experimental conditions. It is important to emphasize that, in the absence of significant system leakage (sealed oronasal mask), all ventilators worked well (data not shown).

It is expected that TFMs cause large air leaks through their built-in orifices, which can sometimes be of a magnitude comparable to that of a patient disconnection. To increase safety, various manufacturers have limited the leak compensation for ICU ventilators to values equal to or lower than 30 L/min (or 0.5 L/s),^(^ ^16^ ^)^ values above which the disconnection alarm of the ventilator goes off. In our study, the mean expiratory leak for the three ventilators that were able to maintain PEEP levels (Servo-i, Vela, and BiPAP Vision) was 45.6 ± 10.8 L/min with the smallest PEEP value (PEEP5), which is greater than when an oronasal mask is used.^(^ ^17^ ^)^ These large air leaks most likely explain why four of the eight ventilators were considered non-operational.

Similar findings have been described previously. Miyoshi et al.^(^ ^11^ ^)^ tested two ICU ventilators and reported that both worked properly with leaks up to 11.3 L/min; however, autotriggering and shutdown of the inspiratory flow occurred with leaks larger than 18 L/min. Another bench study, using an oronasal face mask and three customized leaks, compared nine ICU ventilators equipped with NIV mode and one NIV ventilator (BiPAP Vision).^(^ ^16^ ^)^ When the air leak was increased to 37 L/min, only one ICU ventilator (Servo-i) and the NIV ventilator worked properly without adjustments, whereas four ICU ventilators either went to backup ventilation or were unable to synchronize.^(^ ^16^ ^)^

In our study, the e500 and the Horus ventilators were not able to maintain PEEP values with air leaks close to 30 L/min (or 0.5 L/s). Other authors have recognized the importance of the leak magnitude for PEEP maintenance.^(^ ^18^ ^,^ ^19^ ^)^

Usually, the inspiratory pressurization is evaluated by the PTP within the first 300 ms or 500 ms (PTP300 and PTP500, respectively), because PIF is reached within the first 250-300 ms and the level of pressure support is reached, in most ventilators, within the first 500 ms.^(^ ^20^ ^)^ Due to the magnitude of the air leak found in our study, pressurization was delayed and PTP300 values were very low, rendering this measurement inappropriate for the evaluation of the quality of pressurization. We therefore chose PTP500 and PTPt as the indices of pressurization. Using these variables, we found that the pressurization capacity in the presence of air leaks varied widely among the ventilators, even after reaching the optimal setting of inspiratory pressure slope. This finding is in agreement with those in previous studies.^(^ ^18^ ^,^ ^19^ ^)^

Given the importance of the inspiratory pressure slope on the pressurization capacity, it has been suggested that slope adjustment should be automated for better performance.^(^ ^21^ ^)^ Unexpectedly, the two ventilators with automatic adjustment of slope (e500 and Vela) were outperformed by Servo-i and BiPAP Vision in their ability to maintain pressurization. Nevertheless, it is true that the e500 showed the highest PIF and PIP, and it was the pressure loss during the expiratory phase that hindered its capacity to achieve optimal pressurization.

The trigger delay was smallest in the Servo-i, Vela, and BiPAP Vision. The ventilators that had the greatest difficulty in compensating for air leakage during the expiratory phase (e500 and Horus) were the ones with the highest trigger delays. Leaks in a suboptimally compensated ventilatory system can interfere with the synchrony between the patient and the ventilator because the ventilator relies on monitored pressure and flow in order to trigger each breath, and air leaks can change these signals. Delays of a magnitude similar to our findings were reported in an evaluation of portable ventilators with two pressure levels in a model with small inspiratory leaks (maximum of 0.16 L/s).^(^ ^22^ ^)^

In pressure support mode, the ventilator cycles from inspiration to expiration when the inspiratory flow decreases to a given value or, in most cases, to a proportion of PIF. When the ventilator cannot end the inspiration by this criterion, cycling-off occurs by secondary criteria, usually an upper limit threshold for the inspiratory time.^(^ ^23^ ^)^ In our study, this backup criterion served as the primary mechanism under conditions of high pressures and large air leaks in the Horus, Vela, and e500. Limitations of the cycling-off criteria in ICU ventilators in the presence of air leakage were previously reported,(24) but this problem has been amended in new software versions of some ventilators and new ventilator models^(^ ^25^ ^)^ (see footnotes in Table 1).

Of all tested ICU ventilators, only Servo-i had an acceptable performance with TFM, although it is noteworthy to mention that, in some settings, premature cycling occurred, a finding previously reported regarding this ventilator.(26) The use of TFM with the other ICU ventilators led to considerable asynchrony that would likely cause excessive patient discomfort and noncompliance with NIV. In addition, some ventilators did not offer sufficient assistance to satisfy the demands of the model, and this fact might potentially increase the respiratory muscle load and worsen respiratory failure in a clinical setting.

These findings suggest that, in order to adequately handle the air leaks that occur with the TFM, manufacturers will have to improve the algorithms in ICU ventilators,^(^ ^27^ ^)^ allowing the reset of the upper limit of flow compensation, which is already true for some ventilators (see footnotes in Table 1). Because this type of software change might have safety implications (disconnection alarm), one possible solution would be to implement an adjustable upper limit of flow based on whether the patient is connected to a TFM or not. Another important consequence of air leaks, which can get higher than 1.7 L/s (100 L/min) in some instances, is the extra cost of wasted medical gases, especially with the use of high fractions of inspired oxygen. To overcome this problem, the true need of such large proximal leaks when using TFMs should be investigated as a mathematical model, and bench studies have shown that the dead space of a TFM is smaller than is its internal volume.^(^ ^28^ ^,^ ^29^ ^)^

Recently, new NIV interfaces (PerforMax and Fitlife; Philips Respironics)^(^ ^30^ ^)^ have been marketed with similar characteristics but smaller internal volumes and without the two bores in the mask that act as exhalation valves. These interfaces use two types of elbow: a standard elbow for use with ICU ventilators without air leakage and an entrainment elbow for NIV ventilators. However, it remains unknown whether the use of these masks without leaks (standard elbows) would result in adequate alveolar ventilation even if they were attached to invasive mechanical ventilators (which have separate inspiratory and expiratory limbs). For example, in one report, patients required a high minute ventilation to avoid CO_2_ retention when using a TFM without significant leaks.^(^ ^31^ ^)^ In conditions in which high tidal volumes are considered inappropriate, one possible solution would be to combine the use of the entrainment elbow (with leaks) with invasive mechanical ventilators, thus optimizing CO_2_ washout.

Some limitations of this study should be outlined. First, this is a bench study, and the results should be extrapolated for clinical practice with caution. Second, due to the large air leakage in the system, the ventilators had to generate high inspiratory flows, causing a significant pressure drop, as measured with the pneumotachograph (placed between the ventilator and the proximal pressure transducer), which led to a slight underestimation of the proximal pressures. However, although it was not possible to precisely estimate the absolute values of PEEP, PIP, and PTP produced in the ventilator circuit, it was possible to compare the ventilators. In addition, because the values of PEEP were inferior to the value generated by the ventilator, the expiratory leakage was likely to be a little higher than that measured. Finally, some ventilators or their NIV modes have been updated (see footnotes in Table 1) after the present study was conducted, and these changes might have affected their performances.

In conclusion, due to the large air leaks associated with the use of TFMs, the majority of the ICU ventilators tested were not suitable for NIV with TFMs.
