# Supplementary material for: Performance of ICU ventilators during noninvasive ventilation with large leaks in a total face mask: a bench study
Source: J Bras Pneumol. 2014 May-Jun;40(3):294–303. doi: 10.1590/S1806-37132014000300013 (PMC4109202; doi:10.1590/S1806-37132014000300013)
Supplement: Translation

# Translation: Desempenho de ventiladores de UTI durante ventilação não invasiva com grandes vazamentos em máscara facial total: estudo em simulador mecânico*

**Authors:** Maria Aparecida Miyuki Nakamura, Eduardo Leite Vieira Costa, Carlos Roberto Ribeiro Carvalho, Mauro Roberto Tucci

**Affiliations:** Laboratório de Investigação Médica 09 (LIM 09), Disciplina de Pneumologia, Instituto do Coração, Hospital das Clínicas da Faculdade de Medicina da Universidade de São Paulo, São Paulo (SP) Brasil; UTI Respiratória, Disciplina de Pneumologia, Instituto do Coração, Hospital das Clínicas da Faculdade de Medicina da Universidade de São Paulo; e Pesquisador, Instituto de Ensino e Pesquisa, Hospital Sírio-Libanês, São Paulo (SP) Brasil; Departamento de Cardiopneumologia, Faculdade de Medicina da Universidade de São Paulo, São Paulo (SP) Brasil; Laboratório de Investigação Médica 09 (LIM 09), Disciplina de Pneumologia, Instituto do Coração, Hospital das Clínicas da Faculdade de Medicina da Universidade de São Paulo, São Paulo (SP) Brasil

**Keywords:** Ventiladores mecânicos, Respiração com pressão positiva, Ventilação não invasiva, Segurança de equipamentos, Falha de equipamento, Máscaras

## Abstract

**Objetivo:**

O desconforto e a falta de adaptação às interfaces de ventilação não invasiva (VNI) são obstáculos ao sucesso da VNI. A máscara facial total (MFT) é uma interface de VNI considerada muito confortável. No entanto, devido a seu grande volume interno e, consequentemente, ao aumento da reinalação de CO_2_, a MFT tem orifícios que permitem vazamentos proximais para melhorar a eliminação de CO_2_. É possível que os ventiladores usados na UTI não compensem esse vazamento adequadamente. Neste estudo, buscamos determinar se ventiladores de UTI com módulo de VNI podem ser usados com MFT com grandes vazamentos.

**Métodos:**

Estudo em simulador mecânico conduzido em um laboratório universitário de pesquisa. Oito ventiladores de UTI equipados para realizar VNI e um ventilador específico para VNI foram conectados a uma MFT com grandes vazamentos. Todos foram testados com dois níveis de *positive end-expiratory pressure* (PEEP, pressão expiratória final positiva) e três níveis de pressão de suporte. As variáveis analisadas foram disparo do ventilador, ciclagem, vazamento total e pressurização.

**Resultados:**

Dos oito ventiladores de UTI, quatro não funcionaram (autodisparo ou desligamento inapropriado por detecção incorreta de desconexão), três funcionaram com alguns problemas (valores baixos de PEEP ou grande atraso na ciclagem do ventilador) e apenas um funcionou adequadamente.

**Conclusões:**

A maioria dos ventiladores de UTI testados mostrou-se inadequada para VNI com MFT com grandes vazamentos.

## Introdução

A ventilação não invasiva (VNI) tem sido usada com sucesso no tratamento de insuficiência respiratória de diferentes etiologias.^(^ ^1^ ^)^ A adesão ao tratamento é uma grande preocupação e tem profundo impacto sobre o sucesso da VNI. Na insuficiência respiratória aguda, por exemplo, 40-60% das tentativas de VNI falham em virtude do desconforto da máscara e da não adesão dos pacientes.^(^ ^2^ ^-^ ^4^ ^)^

A máscara facial total (MFT) é uma interface alternativa criada para aumentar a tolerância dos pacientes. A MFT cobre todo o rosto, proporcionando ventilação eficaz por via nasal e oral. Além disso, por meio de uma maior superfície de contacto com a pele, a MFT minimiza o vazamento de gás e ao mesmo tempo evita úlceras por pressão no rosto.^(^ ^5^ ^-^ ^7^ ^)^ No entanto, a MFT tem a desvantagem de ter um volume interno grande (875 mL).^(^ ^8^ ^)^ Portanto, a fim de minimizar a reinalação de CO_2_, a máscara tem dois orifícios de expiração que permitem o escape de ar.^(^ ^5^ ^,^ ^9^ ^,^ ^10^ ^)^ Embora os ventiladores específicos para VNI sejam frequentemente capazes de compensar esses vazamentos de ar intencionais de forma adequada, é possível que os ventiladores de UTI não o sejam.^(^ ^11^ ^)^

A hipótese principal de nosso estudo foi a de que o desempenho dos ventiladores de UTI não é adequado quando são usadas MFT com grandes vazamentos de ar. Avaliamos o desempenho de ventiladores de UTI na VNI por meio de MFT com grandes vazamentos de ar em um modelo laboratorial e comparamos os resultados obtidos com um ventilador mecânico específico para VNI. 

## Métodos

Trata-se de um estudo experimental realizado em 2008 no Laboratório de Investigação Médica 09 (Laboratório de Pneumologia) da Faculdade de Medicina da Universidade de São Paulo, em São Paulo (SP). 

O modelo de VNI (Figura E1, disponível no site do Jornal Brasileiro de Pneumologia: http://www.jornaldepneumologia.com.br/imagebank/images/jbp_v40n3_suplemento.pdf) baseou-se em modelos previamente descritos^(^ ^12^ ^,^ ^13^ ^)^ e consistiu em um pulmão de teste de duas câmaras (TTL 2600; Michigan Instruments, Grand Rapids, MI, EUA) parcialmente conectadas por uma barra de elevação. A primeira câmara (a câmara *drive*) era ligada ao ventilador *drive* e, durante a fase inspiratória, era insuflada, movendo-se juntamente com a segunda câmara (a câmara torácica) e produzindo pressão negativa em seu interior, a qual era transmitida a um segundo pulmão de teste, de uma câmara (Takaoka, São Paulo, Brasil). Esse simulador consiste em um dispositivo de fole em uma caixa rígida; o fole representa o "pulmão", e o espaço entre o fole e a caixa representa o espaço pleural, com comunicação direta com a câmara torácica. O "pulmão" era ligado a uma cabeça de manequim feita de PVC. A MFT (Philips Respironics, Murryville, PA, EUA) era afixada à cabeça do manequim e ligada ao ventilador a ser testado. A complacência do modelo pulmonar mecânico era de 50 mL/cmH_2_O a um volume inspiratório de 500 mL. 

Dois transdutores de pressão (Valydine, Northridge, CA, EUA) eram ligados ao modelo (Figura E1): um para a pressão proximal (entre a máscara e o pneumotacógrafo proximal) e outro para a pressão pleural (entre o pulmão de teste de duas câmaras e o de uma câmara). O fluxo era medido por meio de dois pneumotacógrafos (Hans Rudolph, Kansas City, MO, EUA): um para o fluxo proximal (entre o transdutor de pressão proximal e o conector em forma de Y do circuito do ventilador) e outro para o fluxo distal (entre a via aérea superior do manequim e o pulmão de teste de uma câmara). A resistência do pneumotacógrafo proximal variava com os valores do fluxo. Para fluxos de 0,5 L/s, 1,0 L/s, 2,0 L/s e 3,0 L/s, a resistência era de 1,29 cmH_2_O . L^−1^ . s^−1^, 1,44 cmH_2_O . L^−1^ . s^−1^, 1,91 cmH_2_O . L^−1^ . s^−1^ e 2,40 cmH_2_O . L^−1^ . s^−1^, respectivamente. Os sinais analógicos provenientes dos transdutores eram registrados a 200 Hz e analisados *off-line* por meio de uma versão personalizada do *software* Labview (National Instruments, Austin, TX, EUA). 

Um ventilador Newport e500 (Newport Medical Instruments, Costa Mesa, CA, EUA) foi usado para fornecer ao modelo de VNI o esforço inspiratório. A frequência respiratória foi de 12 min^−1^, no modo de controle de pressão. O tempo inspiratório foi de 1,0 s, a pressão motriz foi de 17 cmH_2_O, a *positive end-expiratory pressure* (PEEP, pressão expiratória final positiva) foi de 0 cmH_2_O e a curva inspiratória foi de +2, gerando um volume corrente de 300 mL no modelo pulmonar mecânico e uma pressão de oclusão de vias aéreas após 0,1 s (P0,1) de 3,4 cmH_2_O.

### Ventiladores testados

Foram testados um ventilador específico para VNI (BiPAP Vision; Philips Respironics) e oito ventiladores de UTI, todos eles equipados para realizar VNI (Tabela E1, disponível no site do Jornal Brasileiro de Pneumologia: http://www.jornaldepneumologia.com.br/imagebank/images/jbp_v40n3_suplemento.pdf): Puritan Bennett 840 (Covidien, Boulder, CO, EUA); Servo-i (Maquet, Solna, Suécia); Vela (Viasys Healthcare, Palm Springs, CA, EUA); Savina (Drägerwerk AG & Co., Lübeck, Alemanha); Esprit (Philips Respironics); GALILEO Gold (Hamilton Medical, Rhäzuns, Suíça); Horus (Taëma, Anthony, França); e e500 (Newport Medical Instruments). 

Quando havia disparo por pressão, este era usado e ajustado para o nível mais sensível que não resultasse em autodisparo. Quando não havia disparo por pressão ou quando o autodisparo era inevitável, usava-se o disparo por fluxo. Sempre que possível, fixava-se o tempo de aumento inspiratório em 50% do valor máximo, o limiar expiratório em 25% do pico de fluxo inspiratório (PFI) e o tempo inspiratório máximo em 1,5 s. As avaliações foram realizadas no modo de pressão de suporte, com PEEP de 5 cmH_2_O (PEEP5) e 10 cmH_2_O (PEEP10), cada qual com três níveis diferentes de pressão de suporte: 5 cmH_2_O (PS5), 10 cmH_2_O (PS10) e 15 cmH_2_O (PS15). Inicialmente, cada ventilador foi testado com uma máscara facial vedada, a fim de verificar o funcionamento da VNI. A MFT foi posteriormente testada com os dois orifícios de expiração abertos para permitir que o ar escapasse, conforme as recomendações do fabricante.^(^ ^5^ ^)^

### Variáveis medidas

Primeiramente, avaliamos se cada ventilador funcionava corretamente com a MFT. O "não funcionamento" do ventilador foi caracterizado por autodisparo constante ou desligamento do fluxo inspiratório (interpretação incorreta de desconexão em virtude de vazamento profuso), mesmo após terem sido experimentadas diferentes configurações de disparo e tempo de aumento inspiratório. Quando o ventilador funcionava, registrávamos os ajustes adicionais necessários para que funcionasse corretamente. 

Medimos as seguintes variáveis (Figura 1): pressão inspiratória proximal (PIP) no fim da fase inspiratória, em cmH_2_O, medida no sensor proximal; PEEP, em cmH_2_O; vazamento inspiratório, em L/s, determinado pela diferença entre o fluxo proximal e o fluxo distal no PFI; vazamento expiratório, em L/s, por meio da medição do fluxo proximal no fim da fase expiratória; PFI, em L/s; volume corrente, em L, calculado por meio da integração do sinal de fluxo proveniente do transdutor de fluxo distal; atraso no disparo, em ms, determinado pelo tempo decorrido entre o início do esforço inspiratório (em pressão pleural) e o início do fluxo inspiratório; atraso na ciclagem, em ms, medido pelo tempo transcorrido desde o fim do esforço inspiratório motor até o fim do fluxo inspiratório do ventilador; produto da pressão e tempo inspiratórios a 500 ms e a 1 s (PPT500 e PPTt, respectivamente), determinado pelo cálculo da área sob a curva de pressão-tempo entre o início do esforço inspiratório para os dois tempos.

**Figura 1:** Representação das variáveis medidas. Traçados de pressão (superior) e fluxo (inferior) de uma medida hipotética com positive end-expiratory pressure (PEEP, pressão expiratória final positiva) de 5 cmH2O e pressão de suporte (PS) de 10cmH2O. No meio, traçado da pressão para o ventilador drive. O traçado superior mostra o produto da pressão e tempo inspiratórios a 500 ms (PPT500; área cinza claro) e a 1 s (PPTt; área cinza escuro mais área cinza claro), ambos expressos em porcentagem de área ideal (áreas hachuradas).

### Análise estatística

Para cada condição experimental, o *software* calculou um ciclo médio representativo por meio da média ponto a ponto de cinco ciclos. Os dados relativos a cada variável são apresentados como um único valor (proveniente do ciclo médio) para cada condição. Os dados relativos às diversas condições são apresentados como média ± dp.

Os ciclos foram muito estáveis, e as variâncias de cada variável foram insignificantes; portanto, em nossa análise, não usamos testes formais de hipótese estatística, mas sim uma comparação nominal dos valores de cada ciclo médio, como em outros estudos semelhantes.^(^ ^13^ ^,^ ^14^ ^)^ Em virtude da baixa variabilidade do modelo mecânico de VNI, qualquer diferença clinicamente relevante nas medidas certamente resultaria em uma diferença de mais de 2,8 vezes o erro técnico de medida (desvio-padrão de vários ciclos de uma condição).(15) Os valores calculados (2,8 vezes o erro técnico de medida) foram os seguintes: volume corrente = 0,020 L; PIP = 0,17 cmH_2_O; PEEP = 0,15 cmH_2_O; PPT500 = 4,3%; PPTt = 2,5%; PFI = 0,05 L/s; vazamento expiratório = 0,03 L/s; atraso no disparo = 9,7 ms e atraso na ciclagem = 7,5 ms. 

## Resultados

O ventilador para VNI e apenas quatro dos ventiladores de UTI equipados para realizar VNI funcionaram com a MFT: Horus, Vela, e500 e Servo-i (Tabela 1). Destes, todos menos o Servo-i tiveram problemas de disparo ou ciclagem. Os principais problemas nos outros ventiladores, que foram considerados não operacionais, foram autodisparo e desligamento indevido em virtude de interpretação incorreta de desconexão. Com a máscara oronasal vedada (isto é, na ausência de vazamento significativo no sistema), todos os ventiladores funcionaram bem.

**Tabela 1:** Desempenho dos ventiladores testadosa com a máscara facial total e os principais problemas observados.

Ventilador	Funcionamento correto	Causa do funcionamento incorreto	Problemas durante o funcionamento
BiPAP Vision	Sim		
Puritan Bennett 840^b,c^	Não	AD	
Savina^b,d^	Não	AD	
GALILEO Gold^b,e^	Não	DFI	
Servo-i^b,f^	Sim		Ciclagem prematura em PEEP5 e PS5 e em PEEP10 e PS5
e500^b,e^	Não	DFI (funcionamento incorreto em PEEP10)	FCV em PEEP5 nos 3 níveis de PS
Esprit^b,g^	Não	AD, DFI	
Horus (VNI desativada)	Não	DFI (funcionamento incorreto em PEEP5)	CTI e FCV em PEEP10 nos 3 níveis de PS
Horus^b,e^	Não	AD	
Vela^b,h^	Não		CTI

VNI : ventilação não invasiva

AD : autodisparo (o ventilador testado manteve uma frequência respiratória maior que a do ventilador *drive* (12 ciclos/min) e o tempo inspiratório foi variável)

DFI : desligamento do fluxo inspiratório (desligamento inadequado após alguns ciclos devido à detecção incorreta de desconexão)

PS5 : pressão de suporte de 5 cmH_2_O

**PEEP5:** : *positive end-expiratory pressure* (pressão expiratória final positiva) de 5 cmH_2_O

**PEEP10:** : *positive end-expiratory pressure* (pressão expiratória final positiva) de 10 cmH_2_O

FCV : falha de compensação de vazamento (o ventilador não é capaz de manter o nível de PEEP)

CTI : ciclagem por tempo inspiratório máximo, de 1,5 s

a BiPAP Vision (Philips Respironics, Murryville, PA, EUA); Puritan Bennett 840 (Covidien, Boulder, CO, EUA); Savina (Drägerwerk AG & Co., Lübeck, Alemanha); GALILEO Gold (Hamilton Medical, Rhäzuns, Suíça); Servo-i (Maquet, Solna, Suécia); e500 (Newport Medical Instruments, Costa Mesa, CA, EUA); Esprit (Philips Respironics); Horus (Taëma, Anthony, França); e Vela (Viasys Healthcare, Palm Springs, CA, EUA)

b Ventilação não invasiva ativada. Atualizações dos ventiladores entre 2008 e 2013

c Puritan Bennett(tm) tem um novo *software* opcional com compensação de vazamentos de até 65 L/min para adultos

d Sem mudanças na compensação de vazamentos. O novo modelo (Savina 300) tem melhor compensação de vazamentos

e Sem atualizações para compensação de vazamentos

f A nova versão do modo de VNI compensa vazamentos de até 65 L/min para adultos

g A nova versão do *software* e o novo modelo (V200) têm compensação de vazamentos de até 60 L/min e disparo e ciclagem autoadaptativos (*autotracking*)

h Compensação de vazamentos de até 40 L/min nos modelos Vela Plus e Vela Comprehensive.

Em todos os ventiladores de UTI, com exceção do Horus, foi necessário acionar o modo de VNI. Para que o Horus, o e500, e o Vela funcionassem em certos contextos, foi necessário ajustar a sensibilidade do disparo expiratório, a curva da pressão inspiratória ou a sensibilidade inspiratória. 

O ventilador Horus não funcionou no modo de VNI. Usando o "modo invasivo" *default*, tivemos de colocá-lo no modo de pressão de suporte, com seu limiar de ciclagem expiratória e curva de pressão inspiratória no máximo (30 L/min e 150 cmH_2_O/s, respectivamente). Não foi possível ajustar corretamente a sensibilidade do disparo inspiratório com a opção de disparo por pressão; só funcionou dentro de uma faixa estreita com a opção de disparo por fluxo (aproximadamente 1,7 L/min). 

A curva de pressão inspiratória do ventilador e500 foi colocada em modo automático, assim como o foi o limiar de ciclagem expiratória (fixado em 50% do pico de fluxo). Para evitar o autodisparo, foi necessário ajustar o disparo por pressão para valores próximos de −3,2 cmH_2_O.

O modo de VNI do ventilador Vela tem uma seleção automática de sensibilidade de disparo inspiratório. O limiar de ciclagem expiratória teve de ser colocado no máximo (30% do pico de fluxo). No entanto, quando a PEEP foi fixada em 10 cmH_2_O e a pressão de suporte foi fixada em 15 cmH_2_O, houve grande atraso na ciclagem inspiratória, criando grandes volumes correntes que excederam a capacidade do modelo pulmonar. 

O ventilador Servo-i funcionou corretamente. Seu modo de VNI tem sensibilidade automática de disparo inspiratório. A curva de pressão inspiratória foi ajustada para 50% do valor máximo (0,2 s), e o limiar de ciclagem expiratória foi fixado em 25% do pico de fluxo. 

A curva do fluxo inspiratório do ventilador para VNI (BiPAP Vision) foi ajustada para 50% do valor máximo (0,2 s). A sensibilidade de disparo inspiratório e expiratório era automática. 

No tocante à PEEP e ao vazamento expiratório, o Servo-i, o Vela e o BiPAP Vision compensaram os vazamentos da MFT durante a expiração, mantendo os valores de PEEP próximos dos valores estabelecidos (Figura 2). A média dos fluxos emitidos por esses ventiladores a fim de compensar os vazamentos durante a expiração foi de 0,65 ± 0,12 L/s para PEEP5 e de 0,89 ± 0,16 L/s para PEEP10. O e500 e o Horus compensaram mal o vazamento e, portanto, não foram capazes de manter a PEEP desejada (Figura 2). Os fluxos máximos de compensação de vazamento para o e500 e o Horus foram, respectivamente, 0,52 ± 0,01 L/s (PEEP5 com a opção de compensação de vazamento ligada) e 0,41 ± 0,01 L/s (PEEP10).

**Figura 2:** Valores medidos de positive end-expiratory pressure (PEEP, pressão expiratória final positiva; painel à esquerda) e fluxo expiratório fornecido pelo ventilador (painel à direita) para compensar o vazamento de ar em PEEP de 5 cmH2O (PEEP5) e 10 cmH2O (PEEP10), na forma de média ± dp. Como se pode observar, os ventiladores Horus e e500 não compensaram adequadamente os vazamentos, fornecendo menos de 0,6 L/s de fluxo compensatório, e não foram capazes de manter o nível estabelecido de PEEP. *Medida não realizada em virtude de autodisparo.

O ventilador Horus teve a menor PIP na máscara (Figura E2, disponível no site do Jornal Brasileiro de Pneumologia: http://www.jornaldepneumologia.com.br/imagebank/images/jbp_v40n3_suplemento.pdf). Todos os outros ventiladores atingiram PIP semelhantes. O BiPAP Vision apresentou os maiores valores de PPT500 e PPTt (Figura 3). O e500 e o Horus apresentaram os menores valores de PPT500.

**Figura 3:** Características de pressurização de cinco dos ventiladores testados, demonstradas pelo produto da pressão e tempo inspiratórios a 500 ms (PPT500; círculos vazados) e a 1 s (PPTt; círculos preenchidos), em porcentagem de área ideal. Os traços horizontais indicam as médias de todas as medidas (pressão expiratória final positiva de 5 e 10 cmH2O vs. pressão de suporte de 5, 10 e 15 cmH2O) disponíveis para os ventiladores.

Os valores de PFI aumentaram paralelamente ao aumento da pressão inspiratória (Figura 4), e o volume dos vazamentos aumentou paralelamente ao aumento da PEEP e da pressão de suporte. A média de PFI para todos os ventiladores foi de 1,69 ± 0,31 L/s em PS5, 2,07 ± 0,26 L/s em PS10 e 2,36 ± 0,31 L/s em PS15. Os maiores PFI foram alcançados pelo BiPAP Vision e pelo e500 (2,39 ± 0,32 L/s e 2,14 ± 0,32 L/s, respectivamente). Os ventiladores Servo-i e Vela apresentaram valores intermediários (2,00 ± 0,32 L/s e 1,82 ± 0,33 L/s, respectivamente), e os menores PFI foram atingidos pelo Horus (1,60 ± 0,29 L/s). 

**Figure 4:** Pico de fluxo inspiratório de cinco dos ventiladores testados. Medidas com positive end-expiratory pressure (PEEP, pressão expiratória final positiva) de 5 cmH2O (PEEP5) e 10 cmH2O (PEEP10) estão à esquerda e à direita, respectivamente. As medições foram feitas em níveis de pressão de suporte de 5, 10 e 15 cmH2O (PS5, PS10 e PS15, respectivamente). *Em virtude de autodisparo, não foram realizadas medidas com o ventilador Horus em PEEP5 nem com o ventilador e500 em PEEP10. #Em virtude da limitação do modelo pulmonar, o ventilador Vela não foi testado com PEEP10 e PS15.

No tocante a volumes correntes, tornaram-se maiores com o aumento dos níveis de pressão de suporte (Figura E3, disponível no site do Jornal Brasileiro de Pneumologia: http://www.jornaldepneumologia.com.br/imagebank/images/jbp_v40n3_suplemento.pdf). Com PEEP5 em PS5, PS10 e PS15, as médias dos volumes correntes para todos os ventiladores foram de 472 ± 25 mL, 609 ± 32 mL e 726 ± 124 mL, respectivamente, ao passo que com PEEP10 em PS5, PS10 e PS15 as médias foram de 489 ± 25 mL, 641 ± 28 mL e 768 ± 90 mL, respectivamente. 

O modelo pulmonar mecânico (sem os ventiladores testados) apresentou um atraso basal intrínseco de 30,5 ms para o atraso no disparo e de 24,9 ms para o atraso na ciclagem. Apenas o e500 e o Horus apresentaram atraso no disparo superior a 100 ms (276 ± 105 ms e 152 ± 36 ms, respectivamente). O Servo-i, o Vela, e o BiPAP Vision, respectivamente, apresentaram atraso no disparo de 50 ± 12 ms, 53 ± 6 ms e 69 ± 27 ms (Figura E4, disponível no site do Jornal Brasileiro de Pneumologia: http://www.jornaldepneumologia.com.br/imagebank/images/jbp_v40n3_suplemento.pdf). Nem a PEEP nem a pressão de suporte alteraram o atraso no disparo. Embora o ventilador Servo-i tenha apresentado o menor atraso na ciclagem (8,5 ± 67 ms), apresentou ciclagem prematura em certos contextos (−94 ms para PEEP5 em PS5 e −43 ms para PEEP10 em PS5). O ventilador BiPAP Vision teve média de atraso na ciclagem de 136 ± 92 ms. O Horus e o Vela apresentaram médias de 273 ± 231 ms e 228 ± 214 ms, respectivamente. Em ambos, por vezes ocorreu ciclagem expiratória por atingirem um tempo inspiratório máximo ajustado de 1,5 s. O ventilador e500 apresentou o maior atraso na ciclagem (590 ± 622 ms; Figura E5, disponível no site do Jornal Brasileiro de Pneumologia: http://www.jornaldepneumologia.com.br/imagebank/images/jbp_v40n3_suplemento.pdf).

## Discussão

O achado mais importante do presente estudo foi o de que apenas um dos ventiladores de UTI testados mostrou-se adequado para VNI com MFT. Dos oito ventiladores de UTI, quatro foram considerados totalmente não operacionais em virtude de desligamento indevido (detecção incorreta de desconexão) ou autodisparo, ao passo que três dos quatro restantes tiveram problemas para compensar os grandes vazamentos através dos orifícios de expiração, resultando em incapacidade de manter a PEEP e a pressão inspiratória, atraso no disparo inspiratório ou atraso na ciclagem de inspiração para expiração. Somente o Servo-i e o ventilador para VNI (BiPAP Vision) funcionaram corretamente em todas as condições experimentais. É importante salientar que, na ausência de vazamento significativo no sistema (máscara oronasal vedada), todos os ventiladores funcionaram bem (dados não mostrados). 

Espera-se que MFT causem grandes vazamentos de ar através de seus orifícios, os quais podem às vezes ser de uma magnitude comparável à de uma desconexão do paciente. Para aumentar a segurança, vários fabricantes têm limitado a compensação de vazamento para ventiladores de UTI a valores iguais ou inferiores a 30 L/min (ou 0,5 L/s),(16) acima dos quais o alarme de desconexão do ventilador dispara. Em nosso estudo, a média de vazamento expiratório para os três ventiladores que foram capazes de manter os níveis de PEEP (Servo-i, Vela e BiPAP Vision) foi de 45,6 ± 10,8 L/min com o menor valor de PEEP (PEEP5), que é maior do que quando uma máscara oronasal é usada.^(^ ^17^ ^)^ Esses grandes vazamentos de ar provavelmente explicam por que quatro dos oito ventiladores foram considerados não operacionais. 

Achados semelhantes foram descritos anteriormente. Miyoshi et al.^(^ ^11^ ^)^ testaram dois ventiladores de UTI e relataram que ambos funcionaram corretamente com vazamentos de até 11,3 L/min; no entanto, autodisparo e desligamento do fluxo inspiratório ocorreram com vazamentos maiores que 18 L/min. Outro estudo experimental, envolvendo máscara oronasal e três vazamentos personalizados, comparou nove ventiladores de UTI equipados para realizar VNI a um ventilador específico para VNI (BiPAP Vision). ^(^ ^16^ ^)^ Quando o vazamento de ar foi aumentado para 37 L/min, apenas um ventilador de UTI (Servo-i) e o ventilador para VNI funcionaram corretamente sem ajustes, ao passo que quatro ventiladores de UTI ou entraram em ventilação de *backup* ou não foram capazes de se sincronizar.^(^ ^16^ ^)^

Em nosso estudo, os ventiladores e500 e Horus não foram capazes de manter os valores de PEEP com vazamentos de aproximadamente 30 L/min (ou 0,5 L/s). Outros autores reconheceram a importância da magnitude do vazamento para a manutenção da PEEP.^(^ ^18^ ^,^ ^19^ ^)^

Geralmente, a pressurização inspiratória é avaliada pelo PPT nos primeiros 300 ms ou 500 ms (PPT300 e PPT500, respectivamente), pois o PFI é atingido nos primeiros 250-300 ms e o nível de pressão de suporte é alcançado, na maioria ventiladores, nos primeiros 500 ms.^(^ ^20^ ^)^ Em virtude da magnitude do vazamento de ar em nosso estudo, houve atraso na pressurização e os valores de PPT300 foram muito baixos, tornando essa medida inadequada para a avaliação da qualidade da pressurização. Portanto, optamos por usar PPT500 e PPTt como índices de pressurização. Usando essas variáveis, constatamos que a capacidade de pressurização na presença de vazamentos de ar variou muito entre os ventiladores, mesmo depois de se atingir a melhor configuração da curva de pressão inspiratória. Esse achado está de acordo com os de estudos anteriores.^(^ ^18^ ^,^ ^19^ ^)^

Dada a importância da curva de pressão inspiratória para a capacidade de pressurização, sugeriu-se que o ajuste da curva deveria ser automático para melhorar o desempenho.^(^ ^21^ ^)^ Inesperadamente, os dois ventiladores com ajuste automático da curva (e500 e Vela) foram superados pelo Servo-i e pelo BiPAP Vision no tocante a sua capacidade de manter a pressurização. Não obstante, é verdade que o e500 apresentou os maiores valores de PFI e PIP, e foi a perda de pressão durante a fase expiratória que prejudicou sua capacidade de atingir a melhor pressurização. 

O atraso no disparo foi menor no Servo-i, no Vela e no BiPAP Vision. Os ventiladores que tiveram a maior dificuldade em compensar o vazamento de ar durante a fase expiratória (e500 e Horus) foram os que apresentaram os maiores atrasos no disparo. Vazamentos em um sistema ventilatório mal compensado podem interferir no sincronismo entre o paciente e o ventilador, pois o ventilador depende de pressão e fluxo monitorados para disparar cada ciclo respiratório, e vazamentos de ar podem alterar esses sinais. Atrasos de uma magnitude semelhante à encontrada em nosso estudo foram relatados em uma avaliação de ventiladores portáteis com dois níveis de pressão em um modelo com pequenos vazamentos inspiratórios (máximo de 0,16 L/s).^(^ ^22^ ^)^

No modo de pressão de suporte, o ventilador faz um ciclo de inspiração a expiração quando o fluxo inspiratório diminui para determinado valor ou, na maioria dos casos, para uma proporção do PFI. Quando o ventilador não consegue terminar a inspiração por esse critério, a ciclagem ocorre por critérios secundários, geralmente um limiar superior para o tempo inspiratório.^(^ ^23^ ^)^ Em nosso estudo, esse critério secundário serviu como o principal mecanismo em condições de altas pressões e grandes vazamentos de ar no Horus, no Vela e no e500. Limitações dos critérios de ciclagem em ventiladores de UTI na presença de vazamento de ar foram previamente relatadas,^(^ ^24^ ^)^ mas esse problema foi corrigido em novas versões de *software* para alguns ventiladores e em novos modelos de ventilador^(^ ^25^ ^)^ (ver as notas de rodapé da Tabela 1). 

De todos os ventiladores de UTI testados, apenas o Servo-i teve um desempenho aceitável com MFT, embora seja importante mencionar que, em alguns contextos, ocorreu ciclagem prematura, um achado já relatado a respeito desse ventilador. ^(^ ^26^ ^)^ O uso de MFT com os outros ventiladores de UTI levou a assincronismo considerável, que provavelmente causaria excessivo desconforto ao paciente e não adesão à VNI. Além disso, alguns ventiladores não ofereceram assistência suficiente para satisfazer as exigências do modelo, e isso pode aumentar a carga dos músculos respiratórios e agravar a insuficiência respiratória em um contexto clínico. 

Esses achados sugerem que, para lidar adequadamente com os vazamentos de ar que ocorrem com o uso de MFT, os fabricantes terão de melhorar os algoritmos em ventiladores de UTI,^(^ ^27^ ^)^ permitindo a redefinição do limite superior de compensação do fluxo, o que já é verdadeiro a respeito de alguns ventiladores (ver as notas de rodapé da Tabela 1). Como esse tipo de mudança no *software* pode ter implicações de segurança (alarme de desconexão), uma possível solução seria a implantação de um limite superior ajustável de fluxo baseado no fato de o paciente estar ou não estar conectado a uma MFT. Outra consequência importante dos vazamentos de ar, que podem ser superiores a 1,7 L/s (100 L/min) em alguns casos, é o custo extra do desperdício de gases medicinais, especialmente com o uso de altas frações inspiradas de oxigênio. Para resolver esse problema, deve-se investigar, na forma de modelo matemático, a verdadeira necessidade de tão grandes vazamentos proximais quando se usa MFT, e estudos experimentais mostraram que o espaço morto de uma MFT é menor do que o seu volume interno.^(^ ^28^ ^,^ ^29^ ^)^

Recentemente, novas interfaces de VNI (PerforMax e Fitlife; Philips Respironics)^(^ ^30^ ^)^ têm sido comercializadas com características semelhantes, porém com volumes internos menores e sem os dois orifícios na máscara, que atuam como válvulas de exalação. Essas interfaces usam dois tipos de cotovelo: um cotovelo-padrão para ser usado com ventiladores de UTI sem vazamento de ar e um cotovelo de expiração para ventiladores específicos para VNI. No entanto, ainda não se sabe se o uso dessas máscaras sem vazamentos (cotovelos-padrão) resultaria em ventilação alveolar adequada, mesmo se fossem conectadas a ventiladores mecânicos invasivos (que têm circuitos inspiratórios e expiratórios separados). Por exemplo, em um relato, os pacientes necessitaram de elevada ventilação minuto a fim de evitar a retenção de CO_2_ quando usaram MFT sem vazamentos significativos.^(^ ^31^ ^)^ Em condições em que altos volumes correntes são considerados inadequados, uma possível solução seria combinar o uso do cotovelo de expiração (com vazamentos) com ventiladores mecânicos invasivos, otimizando assim a eliminação de CO_2_. 

Algumas limitações deste estudo devem ser resumidas aqui. Em primeiro lugar, trata-se de um estudo experimental, e os resultados devem ser extrapolados para a prática clínica com cautela. Em segundo lugar, em virtude do grande vazamento de ar no sistema, os ventiladores precisaram gerar fluxos inspiratórios elevados, provocando significativa queda de pressão, medida por meio do pneumotacógrafo (colocado entre o ventilador e o transdutor de pressão proximal), o que levou a uma ligeira subestimação das pressões proximais. No entanto, embora não tenha sido possível estimar com precisão os valores absolutos de PEEP, PIP e PPT produzidos no circuito dos ventiladores, foi possível comparar os ventiladores. Além disso, como os valores de PEEP foram inferiores ao valor gerado pelo ventilador, é provável que o vazamento expiratório tenha sido um pouco maior do que o medido. Finalmente, alguns ventiladores ou seus modos de VNI foram atualizados (ver as notas de rodapé da Tabela 1) após a realização do presente estudo, e essas mudanças podem ter alterado seu desempenho. 

Em suma, em virtude dos grandes vazamentos de ar associados ao uso de MFT, a maioria dos ventiladores de UTI testados mostrou-se inadequada para VNI com MFT.
